# Associations between fetal or infancy pet exposure and food allergies: The Japan Environment and Children’s Study

**DOI:** 10.1371/journal.pone.0282725

**Published:** 2023-03-29

**Authors:** Hisao Okabe, Koichi Hashimoto, Mika Yamada, Takashi Ono, Kazufumi Yaginuma, Yohei Kume, Mina Chishiki, Akiko Sato, Yuka Ogata, Karin Imaizumi, Tsuyoshi Murata, Hyo Kyozuka, Kosei Shinoki, Seiji Yasumura, Hidekazu Nishigori, Keiya Fujimori, Mitsuaki Hosoya, the Japan Environment and Children’s Study (JECS)

**Affiliations:** 1 Fukushima Regional Center for the Japan Environment and Children’s Study, Fukushima, Japan; 2 Department of Pediatrics, School of Medicine, Fukushima Medical University, Fukushima, Japan; 3 Department of Obstetrics and Gynecology, School of Medicine, Fukushima Medical University, Fukushima, Japan; 4 Department of Public Health, School of Medicine, Fukushima Medical University, Fukushima, Japan; 5 Fukushima Medical Center for Children and Women, Fukushima Medical University, Fukushima, Japan; Kyung Hee University School of Medicine, REPUBLIC OF KOREA

## Abstract

The hygiene hypothesis suggests that pet exposure is effective in preventing allergic disease, and some studies have reported the beneficial effects of dog exposure during fetal development or early infancy on food allergy. However, the effects of exposure to pets other than dogs on the kinds of food allergies remains unaddressed. This study aimed to explore the effect of exposure to various species of pets on the risk of food allergies. We obtained information on pet exposure and food allergy from the Japan Environment and Children’s Study, a nationwide, prospective birth cohort study that included 97,413 mothers and their children. We examined the associations between exposure to various species of pets during fetal development or early infancy and the incidence risk of food allergies. We conducted logistic regression analysis for each pet species, causative food, and timing of exposure. Exposure to dogs or cats during fetal development or early infancy was estimated to reduce the incidence risk of food allergies until the age of 3 years. Dog exposure was estimated to reduce the incidence risk of egg, milk, and nut allergies, and cat exposure was estimated to reduce the incidence risk of egg, wheat, and soybean allergies. However, hamster exposure was estimated to increase the incidence risk of nut allergy. In conclusion, the association between pet exposure and food allergies might differ depending on the pet species and causative food. Continued dog and cat exposure from fetal development to infancy was estimated to reduce the incidence risk of food allergies. The findings of this study shall aid in the design of future studies.

## Introduction

The incidence of food allergies in children has increased over the past few decades, reaching >10% in developed countries [1–3]. Food allergy is a condition that reduces the quality of life of patients and their families, imposes a significant medical cost burden, and is a major trigger of anaphylaxis, which is sometimes fatal [4]. Therefore, preventing its occurrence is a key priority. The notion that early-life exposure to pets or older siblings provides an immunological benefit to human health stems from the hygiene hypothesis, first proposed by Strachan in 1989 and subsequently supported by several epidemiological studies [5–11]. Pet exposure has been suggested to be effective in the prevention of allergic diseases; however, in some developed countries, including Japan, families concerned about allergies continue to avoid owning pets.

In a recent cohort in South Africa, exposure to farm animals during fetal development or infancy decreased the risk of food allergies in the rural population [12]. Some previous studies have reported the beneficial effects of dog exposure during fetal development or early infancy on food allergy in childhood [13–15], while others have reported no statistically significant effects [16]. Additionally, the effect of exposure to pets other than dogs remains unaddressed. To the best of our knowledge, to date, no study has explored the effect of pet exposure on the risk of developing food allergies in Japanese children, and no exhaustive study has explored the association between various species of pets and food allergies. Moreover, the timing of exposure to pets affecting the risk of food allergies in children remains unknown. Pet exposure may affect the development of food allergies through allergen-specific immune pathways; however, large cohorts are required to examine the associations between pet species and causative food types. Therefore, we examined the effects of exposure to various species of pets during fetal development and early infancy on the risk of various kinds of food allergies in Japanese children using data from a nationwide prospective birth cohort study, the Japan Environment and Children’s Study (JECS).

The current study aimed to explore the effects of exposure to various species of pets during fetal development or early infancy on the incidence risk of food allergy in Japanese children. Moreover, the incidence risk of specific food allergies by causative food and the effect of the timing of exposure were explored.

## Materials and methods

### Study design and population

We obtained information regarding pet exposure and food allergies through questionnaires used in the JECS, an ongoing nationwide prospective birth cohort study in Japan funded by the Ministry of the Environment, and its details have been described previously [17–19]. Briefly, the JECS registered 103,060 pregnancies between January 2011 and March 2014. Information was obtained during the first and second/third trimester from the pregnant women using self-administered questionnaires. Detailed information regarding mothers and their children was transcribed from medical records during the first trimester, at delivery, and when the child was 1 month old. After delivery, information was collected at the age of 1 month and then every 6 months via self-report questionnaires completed by caregivers.

We performed statistical analyses using the data set “jecs-ta-20190930 ver008,” which was released in October 2019. The dataset contained the data of 104,062 fetuses linked to the respective maternal data collected until the child was 3 years. Miscarriages, stillbirths, unknown pregnancy outcomes, multiple fetuses, and preterm infants were excluded from the analysis, and we included children with available data on pet exposures, covariates, and food allergies.

The JECS protocol was reviewed and approved by the Ministry of the Environment’s Institutional Review Board on Epidemiological Studies and by the Ethics Committees of all participating institutions. Written informed consent was obtained from all participants. The JECS was conducted in accordance with the tenets of Helsinki Declaration and other nationally valid regulations and guidelines. Authors had not access to information that could identify individual participants during or after data collection.

### Questionnaires and medical records

The questionnaire content, respondents, and response period are presented in S1 Table. Information on the presence of older siblings and maternal history of allergic diseases (bronchial asthma, allergic rhinitis, atopic dermatitis, allergic conjunctivitis, and food allergy) was obtained from the M-T1 questionnaire (answered by pregnant women in the first trimester). Information on pet exposure during gestation, maternal smoking in the second/third trimester, maternal and paternal education, annual household income, and frequency of living room floor cleaning with a vacuum cleaner was obtained from the M-T2 questionnaire (answered by pregnant women in the second/third trimester). Information on the region of residence, maternal use of antibiotics, pregnancy outcome (single or multiple births), maternal age at delivery, delivery mode, infant sex, gestational age, and birth weight was obtained from the medical record transcripts at delivery. Information on feeding type (breastfeeding, formula milk, or both) was obtained from medical record transcripts when the children were 1 month old. Information on attending a childcare facility and pet exposure during early infancy was obtained from the C-6M questionnaire (answered by caregivers when the children were 6 months of age). Information on the prevalence of food allergy was obtained from the C-1Y, C-1hY, C-2Y, and C-3Y questionnaires (answered by caregivers when the children were 1, 1.5, 2, and 3 years old, respectively).

### Pet exposure

We collected information on exposure to all animal species included in the M-T2 questionnaire during the fetal period: dogs kept indoors or outdoors, cats, hamsters, turtles, and birds. We then collected information on exposure during early infancy to all animal species common in the M-T2 and C-6M questionnaires: dogs kept indoors or outdoors and cats.

### Outcomes

The prevalence of food allergy at ages 1, 1.5, 2, and 3 years was assessed based on a parent-reported doctor’s diagnosis obtained from the C-1Y, C-1hY, C-2Y, and C-3Y questionnaires, respectively. We then obtained information on the consumption status of and reaction to specific allergens (egg, milk, wheat, soybean, fish, rice, fruit, crustacean, soba, sesame, and nut) from the C-1hY, C-2Y, and C-3Y questionnaires. We defined the prevalence of specific food allergies (egg, milk, wheat, soybean, fish, rice, fruit, crustacean, soba, sesame, or nut allergy) at ages 1.5, 2, and 3 years as the fulfillment of all of the following conditions at that age: i) avoidance of the specific food; ii) abnormal blood or skin test results for the specific food or allergic symptoms after eating that food; and iii) doctor’s diagnosis of the food allergy. The incidence of food allergies until the age of 3 years was defined as the prevalence of food allergies by the age of 3 years.

### Statistical analysis

First, we examined the association between exposure to various species of pets and the incidence of all food allergies using logistic regression models. Based on clinical experience and previous studies [16,20,21], we assessed the following factors as possible covariables in regression analyses: i) maternal age at delivery (continuous variable); ii) maternal history of allergic diseases (bronchial asthma, allergic rhinitis, atopic dermatitis, allergic conjunctivitis, or food allergy); iii) maternal smoking status during the second/third trimester; iv) highest level of maternal education; v) highest level of paternal education; vi) annual household income during the second/third trimester; vii) presence of older siblings; and viii) region of residence (Hokkaido, Tohoku, Kanto, Chubu, Kinki, Chugoku, Shikoku, or Kyusyu/Okinawa). We checked the collinearity of the selected covariates and included all available covariates in the model without variable selection.

Second, we examined the associations between exposure to various species of pets and the incidence of the aforementioned specific food allergies using logistic regression models with the same covariables.

Third, we divided the participants into four groups according to the timing of exposure to dogs or cats—no-exposure group, fetal exposure group, infantile exposure group, and “both” exposure group (fetal development and early infancy)—to explore the effect of the timing of exposure. We conducted a logistic regression analysis as before, with the no-exposure group as the control.

Statistical analyses were performed using the STATA software (version 17.0; StataCorp, College Station, TX, USA). We evaluated the crude odds ratio (OR) and adjusted OR (aOR) as measures of association and calculated 95% confidence interval (CI) [22,23].

## Results

### Study participants

**Fig 1:**
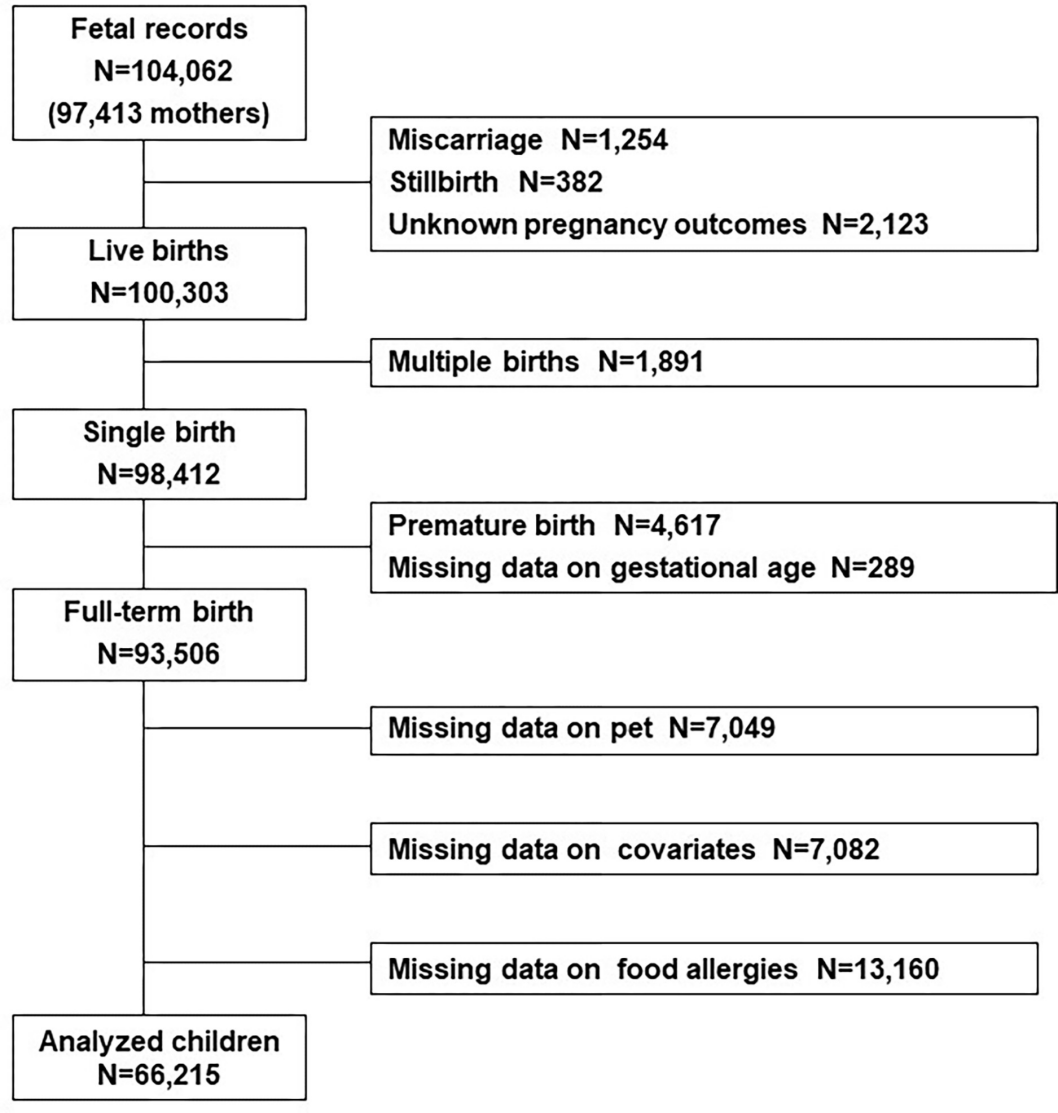
Flowchart of study participant selection.

In the current analysis, we included a total of 66,215 full-term and single-birth children, for whom data on pets, covariates, and food allergies were available (Fig 1). The baseline characteristics of these patients are presented in Table 1. Among them, 21.6% were exposed to pets during the fetal period. The pet-exposed group had higher rates of residents in rural areas (Tohoku and Chugoku) and maternal smoking, more frequent house cleaning with a vacuum cleaner, families with lower educational background and income, and lower rates of presence of older siblings and breastfeeding than the non-exposed group (Table 1).

**Table 1:** Participant characteristics.

	All (N = 66215)	non-exposed (N = 51909)	Any pets exposed (N = 14306)	p-values
Region of residence				
Hokkaido	5313 (8.0%)	4185 (8.1%)	1128 (7.9%)	0.489^‡^
Tohoku	16377 (24.7%)	12302 (23.7%)	4075 (28.5%)	<0.001^‡^
Kanto	8160 (12.3%)	6373 (12.3%)	1787 (12.5%)	0.491^‡^
Chubu	12319 (18.6%)	9982 (19.2%)	2337 (16.3%)	<0.001^‡^
Kinki	8891 (13.4%)	7143 (13.8%)	1748 (12.2%)	<0.001^‡^
Chugoku	1998 (3.0%)	1530 (2.9%)	468 (3.3%)	0.045^‡^
Shikoku	4333 (6.5%)	3396 (6.5%)	937 (6.5%)	0.974^‡^
KyushuOkinawa	8824 (13.3%)	6998 (13.5%)	1826 (12.8%)	0.025^‡^
Maternal history of allergic disease	33487 (50.6%)	26385 (50.8%)	7102 (49.6%)	0.012^‡^
Maternal smoking at the second trimester	2135 (3.2%)	1389 (2.7%)	746 (5.2%)	<0.001^‡^
Cleaning with a vacuum cleaner everyday	11101 (16.8%)	8228 (15.9%)	2873 (20.1%)	<0.001^‡^
Maternal highest level of education:				
Junior high school	2151 (3.2%)	1401 (2.7%)	750 (5.2%)	<0.001^‡^
High school	19140 (28.9%)	14055 (27.1%)	5085 (35.5%)	<0.001^‡^
Technical/vocational college, etc.*	28776 (43.5%)	22747 (43.8%)	6029 (42.1%)	<0.001^‡^
Bachelor’s degree or above†	16148 (24.4%)	13706 (26.4%)	2442 (17.1%)	<0.001^‡^
Paternal highest level of education:				
Junior high school	3929 (5.9%)	2613 (5.0%)	1316 (9.2%)	<0.001^‡^
High school	23132 (34.9%)	17265 (33.3%)	5867 (41.0%)	<0.001^‡^
Technical/vocational college, etc.*	15216 (23.0%)	11909 (22.9%)	3307 (23.1%)	0.661^‡^
Bachelor’s degree or above†	23938 (36.2%)	20122 (38.8%)	3816 (26.7%)	<0.001^‡^
Annual household income (Japanese yen):				
<4000000	25347 (38.3%)	19229 (37.0%)	6118 (42.8%)	<0.001^‡^
4000000 to <8000000	33382 (50.4%)	26815 (51.7%)	6567 (45.9%)	<0.001^‡^
≥8000000	7486 (11.3%)	5865 (11.3%)	1621 (11.3%)	0.914^‡^
Use of antibacterial drugs during pregnancy	13887 (21.0%)	10759 (20.7%)	3128 (21.9%)	0.003^‡^
Maternal age (years)	32 [28–35]	32 [28–35]	31 [28–35]	<0.001
Males	33558 (50.7%)	26289 (50.6%)	7269 (50.8%)	0.724^‡^
Gestational age (weeks)	39 [38–40]	39 [38–40]	39 [38–40]	0.8638^§^
Birth weight (g)	3046 [2814–3294]	3046 [2815–3292]	3048 [2814–3296]	0.916^§^
Cesarean delivery	11497 (17.4%)	8861 (17.1%)	2636 (18.5%)	<0.001^‡^
Elder sibling	35771 (54.0%)	28514 (54.9%)	7257 (50.7%)	<0.001^‡^
Methods of feeding:				
Breastfeeding	36288 (56.7%)	28998 (57.9%)	7290 (52.7%)	<0.001^‡^
Mixed feeding	25673 (40.1%)	19674 (39.3%)	5999 (43.3%)	<0.001^‡^
Infant formula	1983 (3.1%)	1427 (2.8%)	556 (4.0%)	<0.001^‡^
Childcare facility at 6th month	4247 (6.4%)	3206 (6.2%)	1041 (7.3%)	<0.001^‡^
Incidents of atopic dermatitis	5973 (11.2%)	4673 (11.2%)	1300 (11.3%)	0.651^‡^
Exposure during fetal				
Any species of pet	14306 (21.6%)			
Dog kept inside of residence	5724 (8.6%)			
Dog kept outside residence	2531 (3.8%)			
Ct	4209 (6.4%)			
Turtle	742 (1.1%)			
Hamster	604 (0.9%)			
Bird	476 (0.7%)			
Exposure during early infancy				
Dog kept inside residence	6933 (10.5%)	1487 (2.9%)	5446 (38.1%)	
Dog kept outside of residence	2906 (4.4%)	649 (1.3%)	2257 (15.8%)	
Cat	5092 (7.7%)	1106 (2.1%)	3986 (27.9%)	
Prevalence of food allergy at 1.5 years of age	6350 (9.6%)	5115 (9.9%)	1235 (8.6%)	
Prevalence of food allergy at 2 years of age	4399 (6.6%)	3548 (6.8%)	851 (6.0%)	
Prevalence of food allergy at 3 years of age	3908 (5.9%)	3139 (6.1%)	769 (5.4%)	

*Technical junior college, Technical/vocational college, Associate degree.

†Bachelor’s degree, Graduate degree (Master’s/Doctor’s).

‡Categorical variables were tested with the chi-square test.

§Continuous variables were tested with the t-test.

### Effect of pet exposure on the incidence of all food allergies until 3 years of age

**Fig 2:**
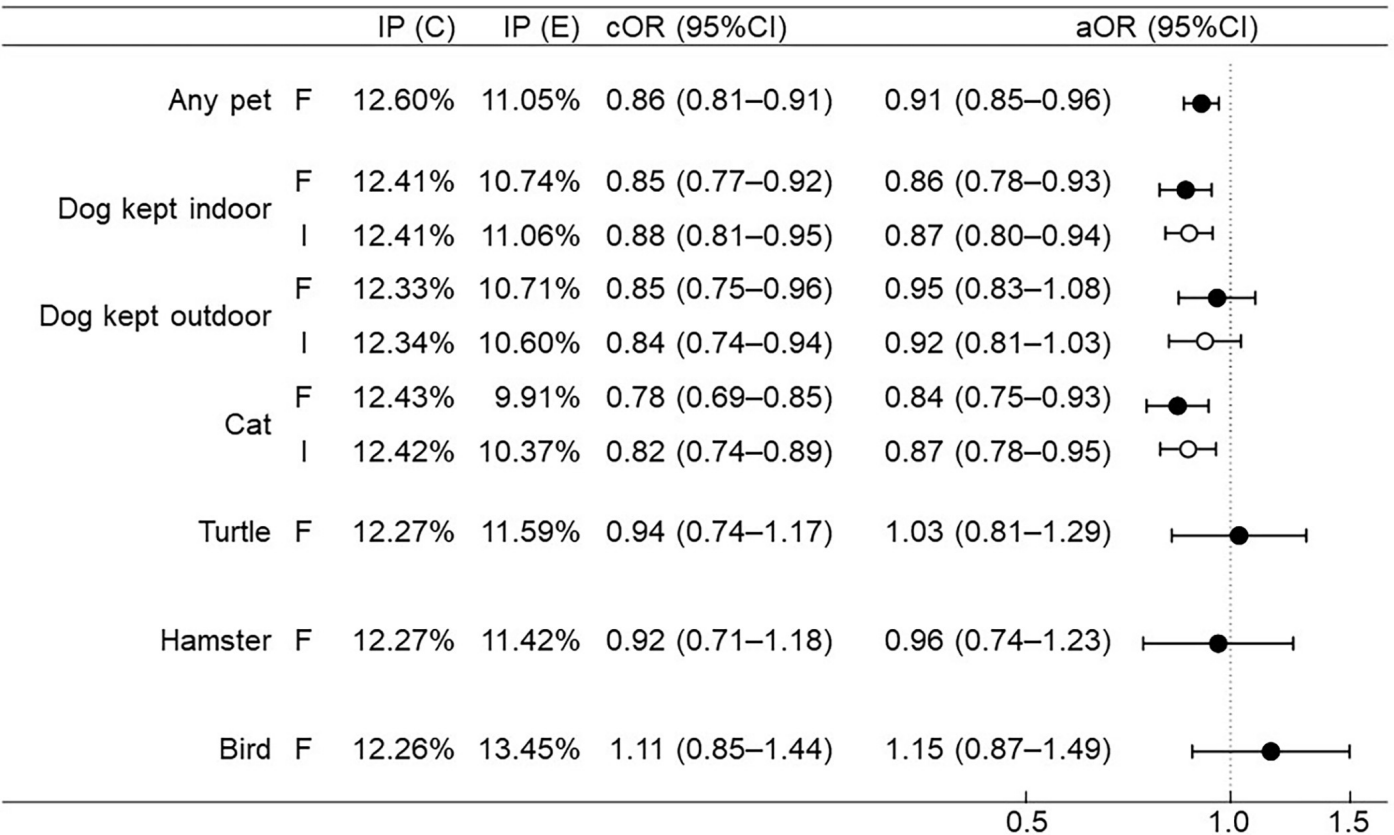
Association between pet exposure and the incidence risk of food allergies until 3 years of age. F, fetal (black circles); I, during early infancy (white circles); IP (C), incidence proportion (control); IP (E), incidence proportion (exposed); cOR, crude odds ratio; aOR, adjusted odds ratio; CI, confidence interval.

No multicollinearity was identified for the selected covariates (S2 Table). Exposure to dogs kept indoors or cats during fetal development or early infancy was estimated to reduce the incidence risk of all food allergies until the age of 3 years (aORs [95% CIs] for dogs kept indoors during fetal development, 0.86 [0.78–0.93]; dogs kept indoors during early infancy, 0.87 [0.80–0.94]; cats during fetal development, 0.84 [0.75–0.93]; cats during early infancy, 0.87 [0.78–0.95]). Crude analysis estimated that exposure to dogs kept outdoors also reduced the incidence risk of all food allergies until the age of 3 years, but after adjusting for covariates, no statistical significance was identified. No significant associations between exposure to turtles, hamsters, and birds during fetal development and the incidence of all food allergies until the age of 3 years were uncovered (Fig 2).

### Effect of pet exposure on the incidence risk of specific food allergies until 3 years of age

#### Egg allergy

**Fig 3:**
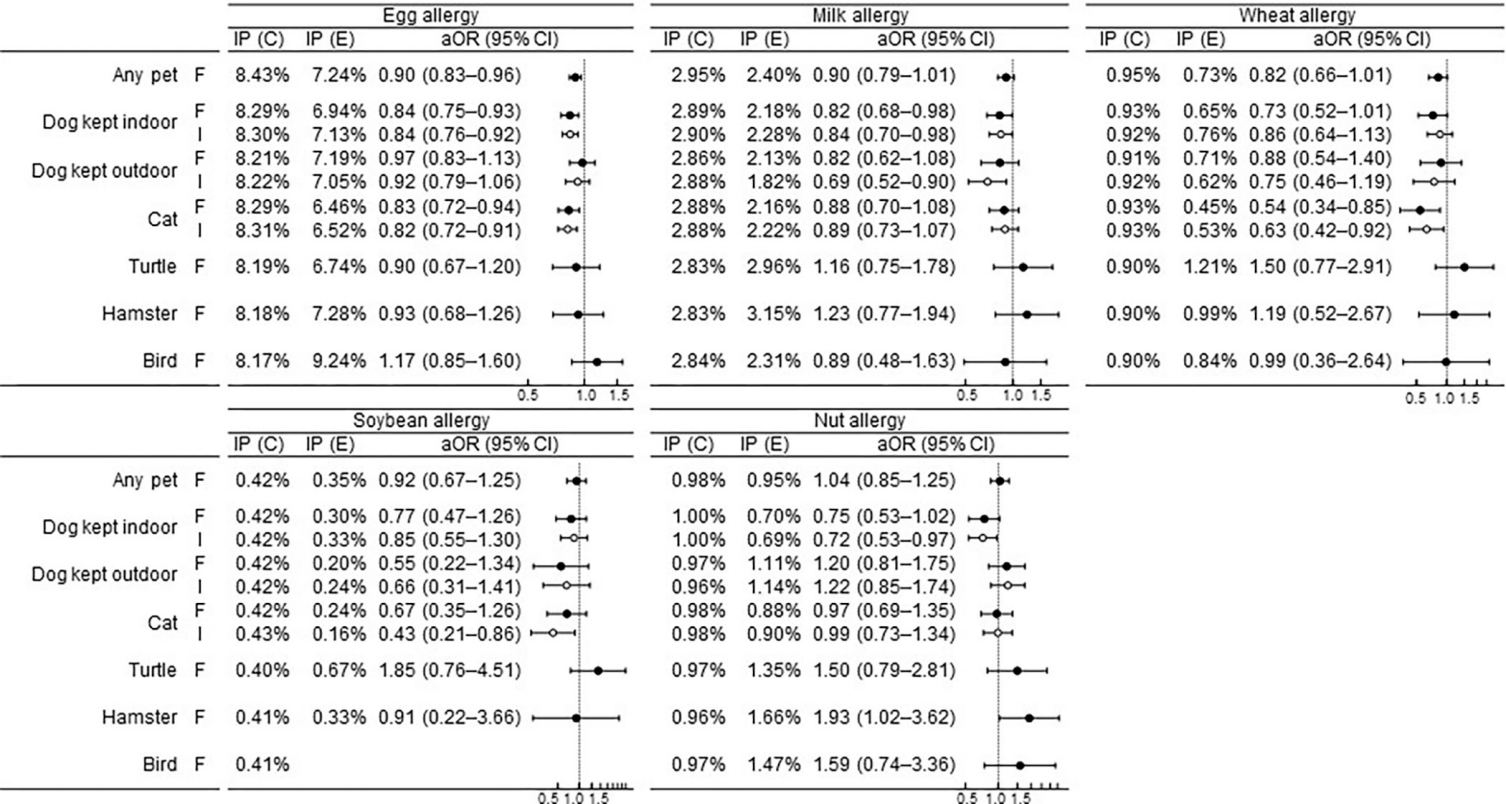
Association between pet exposure and the incidence risk of specific food allergies until 3 years of age. F, fetal (black circles); I, during early infancy (white circles); IP (C), incidence proportion (control); IP (E), incidence proportion (exposed); cOR, crude odds ratio; aOR, adjusted odds ratio; CI, confidence interval.

The incidence risk of egg allergy until the age of 3 years was estimated to be reduced by exposure to dogs kept indoors during fetal development (aORs [95% CIs] 0.84 [0.75–0.93]): to dogs kept indoors during early infancy (0.84 [0.76–0.92]); to cats during fetal development (0.83 [0.72–0.94]); and to cats during early infancy (0.82 [0.72–0.91]) (Fig 3).

#### Milk allergy

The incidence risk of milk allergy until the age of 3 years was estimated to be reduced by exposure to dogs kept indoors during fetal development (aORs [95% CIs] 0.82 [0.68–0.98]): to dogs kept indoors during early infancy (aORs [95% CIs] 0.84 [0.70–0.98]); to cats during fetal development (0.83 [0.72–0.94]); and to dogs kept outdoors during early infancy (0.69 [0.52–0.90]) (Fig 3).

#### Wheat allergy

The incidence risk of wheat allergy until the age of 3 years was estimated to be reduced by exposure to cats during fetal development (aORs [95% CIs] 0.54 [0.34–0.85]): during early infancy (aORs [95% CIs] 0.84 [0.70–0.98]); to cats during fetal development (0.63 [0.42–0.92]) (Fig 3).

#### Soybean allergy

The incidence risk of soybean allergy until the age of 3 years was estimated to be reduced by exposure to cats during early infancy (aORs [95% CIs] 0.43 [0.21–0.86]) (Fig 3).

#### Nut allergy

The incidence risk of nut allergy until the age of 3 years was estimated to be reduced by exposure to dogs kept indoors during early infancy (aORs [95% CIs] 0.72 [0.53–0.97]), while to be increased by exposure to hamsters during fetal development (1.93 [1.02–3.62]) (Fig 3).

#### Other specific food allergies

Fish, fruit, crustacean, and soba allergies showed no significant differences in association with exposure to any pet species. The number of rice and sesame allergy cases was small and could not be adequately adjusted for covariates (S2 Table).

#### Other pet species

No significant differences were found in the associations between exposure to turtles or birds and the incidence of any specific food allergies until the age of 3 years.

### Association between the timing of pet exposure and incidence risk of food allergies

There were 51,858 children in the non-exposure group, 1,072 in the fetal exposure group, 3,129 in the infantile exposure group, and 10,156 in the “both” exposure group. We conducted logistic regression analysis on the incidence of food, egg, milk, wheat, soybean, and nut allergies until the age of 3 years. The incidence risk of food allergy was estimated to be reduced by pet exposure during fetal development and early infancy, but not during only one. Similarly, reduction in the incidence risks of milk, wheat, and soybean allergies required exposure during both times, whereas egg allergy required exposure during early infancy or both stages but not during fetal development alone. Nut allergy was not significantly associated with dog or cat exposure at any time. Dog or cat exposure during fetal development alone did not have a significant effect on the incidence risk of food allergies (Table 2).

**Table 2:** Association between the timing of dog or cat exposure and the incidence risk of food allergies.

Dog or cat exposure	All food allergies			Egg allergy		
	IP	aOR	(95% CI)	IP	aOR	(95% CI)
No-exposure	12.61%			8.45%		
Fetal exposure	12.22%	1.00	(0.82–1.20)	8.68%	1.06	(0.85–1.31)
Infantile exposure	12.02%	0.92	(0.81–1.02)	7.70%	0.86	(0.75–0.98)
Both exposure	10.59%	0.87	(0.80–0.93)	6.83%	0.84	(0.77–0.91)
	**Milk allergy**			**Wheat allergy**		
	**IP**	**aOR**	**(95% CI)**	**IP**	**aOR**	**(95% CI)**
No-exposure	2.99%			0.96%		
Fetal exposure	2.71%	1.03	(0.70–1.49)	0.93%	1.06	(0.56–1.99)
Infantile exposure	2.30%	0.81	(0.63–1.03)	0.96%	1.03	(0.71–1.49)
Both exposure	2.21%	0.83	(0.71–0.95)	0.58%	0.66	(0.49–0.86)
	**Soybean allergy**			**Nut allergy**		
	**IP**	**aOR**	**(95% CI)**	**IP**	**aOR**	**(95% CI)**
No-exposure	0.44%			1.01%		
Fetal exposure	0.56%	1.46	(0.64–3.30)	0.56%	0.61	(0.26–1.35)
Infantile exposure	0.35%	0.85	(0.46–1.57)	0.80%	0.82	(0.54–1.23)
Both exposure	0.25%	0.64	(0.42–0.96)	0.89%	0.94	(0.74–1.17)

IP, incidence proportion; aOR, adjusted odds ratio; CI, confidence interval.

## Discussion

We examined the effects of exposure to various pet species during fetal development and early infancy on the risk of food allergies in Japanese children, using data from the JECS. Our findings suggest that exposure to dogs and cats might be beneficial against the development of certain food allergies, thereby alleviating concerns about pet keeping and reducing the burden of food allergies.

Gern et al. reported that there was no significant effect of cat or dog ownership at birth on food allergy at the age of 1 year [16]. In contrast, Marrs et al. reported that dog exposure during infancy decreases the incidence risk of food allergy at the age of 1–3 years [14], and Smejda et al. argued that dog exposure before and during pregnancy decreases the risk of food allergy during the first year of life [15]; these findings are consistent with our results. However, no significant difference in the association between the incidence risk of food allergies and cat exposure was reported in these studies [14–16], contrary to our results. The difference in sample size (much smaller in previous studies) is the most likely reason for this discrepancy. Marrs et al. reported an aOR (95% CI) of 0.59 (0.26–1.30) for cat exposure during infancy [14], suggesting that a larger sample size might have produced results similar to ours. The fact that Smejda et al. investigated cat exposure before delivery and not during infancy might be a factor for the different results from those of our study. In addition, differences in pet-keeping environments in these countries may have affected the results. Kopline et al. found that having dogs indoors was inversely associated with egg allergy at 1 year of age, which is consistent with our results. However, no significant association between the incidence risk of egg allergy and cat exposure was observed [13]. Our study, with a larger sample size, showed significant results regarding cats. Peter et al. reported from the HealthNuts study that having dogs indoors was inversely associated with multiple food allergies (predominantly to peanuts) at the age of 1 year, which is consistent with our results. However, the HealthNuts study did not consider nut allergies other than peanut allergy and did not examine exposure to pets other than dogs and cats [24]. To the best of our knowledge, we report, for the first time, associations between hamster exposure and nut allergy, dog exposure and milk allergy, as well as cat exposure and wheat and soybean allergies. Smejda et al. also studied the association of pet exposure before and during pregnancy with food allergies, and Koplin et al., Marrs et al., and Perers et al. investigated exposure during infancy [13–15,24]. However, they did not investigate exposure during only fetal development or infancy, and it is unclear what time of pet exposure affects food allergy. We found that dog or cat exposure only during the fetal period have no significant effect on the incidence risk of food allergies. This finding suggests that continued dog or cat ownership after birth may be beneficial in reducing the risk of food allergies.

Although the mechanisms by which pet exposure affect food allergies have not yet been elucidated, several hypotheses have been postulated. The first is related to the gut microbiota. Pet exposure can affect the infant’s gut microbiota directly or indirectly through changes in the parent’s gut microbiota or home microbiome [25–28]. Fujimura et al. reported that the state of an infant’s gut microbiota might affect CD4^+^ cells and induce IgE sensitization [29]. Tun et al. reported that pet exposure increased the abundance of two bacteria, Ruminococcus and Oscillospira, which are negatively associated with childhood atopy [30]. The second is the endotoxin-mediated mechanism. Pet ownership reportedly increases endotoxin levels at home, which may protect against allergen sensitization by enhancing type 1 immunity [31,32]. The third is the skin barrier-mediated mechanism. Atopic dermatitis is a major risk factor for the incidence of food allergies because of the disruption of the skin barrier function and increased susceptibility to percutaneous sensitization [33,34]. Pet exposure has been reported to affect the risk of atopic dermatitis [16,35]. These mechanisms may explain the suppressive effect of dog and cat exposures on the food allergies observed in the present study. However, the increased incidence risk of nut allergy with hamster exposure may be explained by the fact that hamsters feed on nuts. In other words, we assumed that nut allergens can percutaneously sensitize infants through physical contact or house dust. Therefore, family hand washing and keeping hamsters away from babies might minimize the risk of nut allergy even if hamsters are kept as pets.

We also demonstrated the benefits of maintaining dogs and cats after birth. These findings reduce concerns about the development of allergic diseases caused by keeping dogs and cats. Reducing the incidence of food allergies will significantly reduce childhood mortality from anaphylaxis.

The strengths of this study include the use of a large sample from the general population across Japan, the longitudinal design, and the inclusion of various factors. To the best of our knowledge, this is the largest birth cohort study on the association between pet exposure and food allergy risk. The longitudinal design minimized the possibility of causal reversal. In addition, most potentially confounding information was covered, allowing for adjustment for covariates.

However, some limitations should be noted. First, we performed no objective evaluation, such as oral food challenge, blood tests, or skin tests, to diagnose a food allergy. Instead, we relied on a parent-reported doctor’s diagnosis obtained from questionnaires. Although oral food challenge is the best method for diagnosing food allergies, it carries the risk of inducing anaphylaxis and is not always adequate for large cohort studies. The blood/skin test indicates sensitization to the allergen, but many people can eat the food even if sensitized. The outcomes we defined well reflect food consumption status that impacts the quality of life. Second, recall bias is a major concern in birth cohorts with self-report questionnaires, leading to underestimation of the incidence of food allergies. However, frequent questionnaires are administered every 6 months after birth in the JECS. Third, we could not obtain information on the gut microbiota or endotoxin levels and could not explore the underlying mechanisms of food allergy. Detailed prospective studies including such information or basic research will be necessary to elucidate these mechanisms. Fourth, various factors can confound the association between pet exposure and food allergies. Potential confounding factors may not be entirely regulated. Only randomized controlled trials prospectively assigning pet exposure could completely remove potential confounding factors. However, such studies would likely pose challenging ethical issues.

## Conclusion

This study showed that the association between pet exposure during fetal development or early infancy and the incidence risk of food allergies until the age of 3 years differs depending on the combination of two factors: pet species and allergen type. Dog exposure might reduce the incidence risks of egg, milk, and nut allergies; cat exposure might reduce the risks of egg, wheat, and soybean allergies; hamster exposure might increase the risk of nut allergy. However, this study was a questionnaire-based survey, and we did not perform an objective assessment. Further studies using oral food challenges are required to more accurately assess the incident of food allergies. Nevertheless, the findings of this study shall aid in the design of future studies.

## Supporting information

S1 Table Questionnaire content. (DOCX)

S2 Table Collinearity of the selected covariates. (DOCX)

S3 Table Supplementary material. (DOCX)

## References

[1] Willits EK, Park MA, Hartz MF, Schleck CD, Weaver AL, Joshi AY. Food Allergy: A Comprehensive Population-Based Cohort Study. Mayo Clin Proc. 2018;93(10):1423–30. doi: 10.1016/j.mayocp.2018.05.031 ; PubMed Central PMCID: PMC6366995. 30286830 PMC6366995

[2] Branum AM, Lukacs SL. Food allergy among children in the United States. Pediatrics. 2009;124(6):1549–55. Epub 20091116. doi: 10.1542/peds.2009-1210 . 19917585

[3] Osborne NJ, Koplin JJ, Martin PE, Gurrin LC, Lowe AJ, Matheson MC, et al. Prevalence of challenge-proven IgE-mediated food allergy using population-based sampling and predetermined challenge criteria in infants. J Allergy Clin Immunol. 2011;127(3):668–76.e1-2. doi: 10.1016/j.jaci.2011.01.039 . 21377036

[4] Grabenhenrich LB, Dölle S, Moneret-Vautrin A, Köhli A, Lange L, Spindler T, et al. Anaphylaxis in children and adolescents: The European Anaphylaxis Registry. J Allergy Clin Immunol. 2016;137(4):1128–37.e1. Epub 20160121. doi: 10.1016/j.jaci.2015.11.015 . 26806049

[5] Strachan DP. Hay fever, hygiene, and household size. BMJ. 1989;299(6710):1259–60. doi: 10.1136/bmj.299.6710.1259 ; PubMed Central PMCID: PMC1838109. 2513902 PMC1838109

[6] Litonjua AA, Milton DK, Celedon JC, Ryan L, Weiss ST, Gold DR. A longitudinal analysis of wheezing in young children: the independent effects of early life exposure to house dust endotoxin, allergens, and pets. J Allergy Clin Immunol. 2002;110(5):736–42. doi: 10.1067/mai.2002.128948 . 12417882

[7] Ownby DR, Johnson CC, Peterson EL. Exposure to dogs and cats in the first year of life and risk of allergic sensitization at 6 to 7 years of age. JAMA. 2002;288(8):963–72. doi: 10.1001/jama.288.8.963 . 12190366

[8] Takkouche B, González-Barcala FJ, Etminan M, Fitzgerald M. Exposure to furry pets and the risk of asthma and allergic rhinitis: a meta-analysis. Allergy. 2008;63(7):857–64. doi: 10.1111/j.1398-9995.2008.01732.x . 18588551

[9] Lodge CJ, Allen KJ, Lowe AJ, Hill DJ, Hosking CS, Abramson MJ, et al. Perinatal cat and dog exposure and the risk of asthma and allergy in the urban environment: a systematic review of longitudinal studies. Clin Dev Immunol. 2012;2012:176484. Epub 20111130. doi: 10.1155/2012/176484 ; PubMed Central PMCID: PMC3251799. 22235226 PMC3251799

[10] Fall T, Lundholm C, Örtqvist AK, Fall K, Fang F, Hedhammar Å, et al. Early exposure to dogs and farm animals and the risk of childhood asthma. JAMA Pediatr. 2015;169(11):e153219. Epub 20151102. doi: 10.1001/jamapediatrics.2015.3219 . 26523822

[11] Wegienka G, Havstad S, Kim H, Zoratti E, Ownby D, Woodcroft KJ, et al. Subgroup differences in the associations between dog exposure during the first year of life and early life allergic outcomes. Clin Exp Allergy. 2017;47(1):97–105. Epub 20161010. doi: 10.1111/cea.12804 ; PubMed Central PMCID: PMC5195869. 27562398 PMC5195869

[12] Levin ME, Botha M, Basera W, Facey-Thomas HE, Gaunt B, Gray CL, et al. Environmental factors associated with allergy in urban and rural children from the South African Food Allergy (SAFFA) cohort. J Allergy Clin Immunol. 2020;145(1):415–26. Epub 20191010. doi: 10.1016/j.jaci.2019.07.048 . 31606483

[13] Koplin JJ, Dharmage SC, Ponsonby AL, Tang ML, Lowe AJ, Gurrin LC, et al. Environmental and demographic risk factors for egg allergy in a population-based study of infants. Allergy. 2012;67(11):1415–22. Epub 20120907. doi: 10.1111/all.12015 . 22957661

[14] Marrs T, Logan K, Craven J, Radulovic S, McLean W, Lack G, et al. Dog ownership at three months of age is associated with protection against food allergy. Allergy. 2019;74(11):2212–9. Epub 20190708. doi: 10.1111/all.13868 . 31077604

[15] Smejda K, Polanska K, Stelmach W, Majak P, Stelmach I. Dog keeping at home before and during pregnancy decreased the risk of food allergy in 1-year-old children. Postepy Dermatol Alergol. 2020;37(2):255–61. Epub 20200506. doi: 10.5114/ada.2018.80584 ; PubMed Central PMCID: PMC7262798. 32489363 PMC7262798

[16] Gern JE, Reardon CL, Hoffjan S, Nicolae D, Li Z, Roberg KA, et al. Effects of dog ownership and genotype on immune development and atopy in infancy. J Allergy Clin Immunol. 2004;113(2):307–14. doi: 10.1016/j.jaci.2003.11.017 . 14767447

[17] Michikawa T, Nitta H, Nakayama SF, Yamazaki S, Isobe T, Tamura K, et al. Baseline profile of participants in the Japan environment and children’s study (JECS). J Epidemiol. 2018;28(2):99–104. Epub 20171025. doi: 10.2188/jea.JE20170018 ; PubMed Central PMCID: PMC5792233. 29093304 PMC5792233

[18] Kawamoto T, Nitta H, Murata K, Toda E, Tsukamoto N, Hasegawa M, et al. Rationale and study design of the Japan environment and children’s study (JECS). BMC Public Health. 2014;14:25. Epub 20140110. doi: 10.1186/1471-2458-14-25 ; PubMed Central PMCID: PMC3893509. 24410977 PMC3893509

[19] Michikawa T, Nitta H, Nakayama SF, Ono M, Yonemoto J, Tamura K, et al. The Japan Environment and Children’s Study (JECS): a preliminary report on selected characteristics of approximately 10 000 pregnant women recruited during the first year of the study. J Epidemiol. 2015;25(6):452–8. Epub 20150425. doi: 10.2188/jea.JE20140186 ; PubMed Central PMCID: PMC4444500. 25912098 PMC4444500

[20] Almqvist C, Egmar AC, van Hage-Hamsten M, Berglind N, Pershagen G, Nordvall SL, et al. Heredity, pet ownership, and confounding control in a population-based birth cohort. J Allergy Clin Immunol. 2003;111(4):800–6. doi: 10.1067/mai.2003.1334 . 12704361

[21] Eller E, Roll S, Chen CM, Herbarth O, Wichmann HE, von Berg A, et al. Meta-analysis of determinants for pet ownership in 12 European birth cohorts on asthma and allergies: a GA2LEN initiative. Allergy. 2008;63(11):1491–8. doi: 10.1111/j.1398-9995.2008.01790.x . 18721248

[22] Lee SW. Methods for testing statistical differences between groups in medical research: statistical standard and guideline of Life Cycle Committee. Life Cycle. 2022;2:e1. doi: 10.54724/lc.2022.e1

[23] Lee SW. Regression analysis for continuous independent variables in medical research: statistical standard and guideline of Life Cycle Committee. Life Cycle. 2022;2:e3. doi: 10.54724/lc.2022.e3

[24] Peters RL, Allen KJ, Dharmage SC, Lodge CJ, Koplin JJ, Ponsonby AL, et al. Differential factors associated with challenge-proven food allergy phenotypes in a population cohort of infants: a latent class analysis. Clin Exp Allergy. 2015;45(5):953–63. doi: 10.1111/cea.12478 . 25523199

[25] Azad MB, Konya T, Maughan H, Guttman DS, Field CJ, Sears MR, et al. Infant gut microbiota and the hygiene hypothesis of allergic disease: impact of household pets and siblings on microbiota composition and diversity. Allergy Asthma Clin Immunol. 2013;9(1):15. Epub 20130422. doi: 10.1186/1710-1492-9-15 ; PubMed Central PMCID: PMC3655107. 23607879 PMC3655107

[26] Nermes M, Endo A, Aarnio J, Salminen S, Isolauri E. Furry pets modulate gut microbiota composition in infants at risk for allergic disease. J Allergy Clin Immunol. 2015;136(6):1688–90.e1. Epub 20150903. doi: 10.1016/j.jaci.2015.07.029 . 26343452

[27] Konya T, Koster B, Maughan H, Escobar M, Azad MB, Guttman DS, et al. Associations between bacterial communities of house dust and infant gut. Environ Res. 2014;131:25–30. Epub 20140315. doi: 10.1016/j.envres.2014.02.005 . 24637181

[28] Sitarik AR, Havstad S, Levin AM, Lynch SV, Fujimura KE, Ownby DR, et al. Dog introduction alters the home dust microbiota. Indoor Air. 2018;28(4):539–47. Epub 20180313. doi: 10.1111/ina.12456 ; PubMed Central PMCID: PMC6003855. 29468742 PMC6003855

[29] Fujimura KE, Sitarik AR, Havstad S, Lin DL, Levan S, Fadrosh D, et al. Neonatal gut microbiota associates with childhood multisensitized atopy and T cell differentiation. Nat Med. 2016;22(10):1187–91. Epub 20160912. doi: 10.1038/nm.4176 ; PubMed Central PMCID: PMC5053876. 27618652 PMC5053876

[30] Tun HM, Konya T, Takaro TK, Brook JR, Chari R, Field CJ, et al. Exposure to household furry pets influences the gut microbiota of infant at 3–4 months following various birth scenarios. Microbiome. 2017;5(1):40. Epub 20170406. doi: 10.1186/s40168-017-0254-x ; PubMed Central PMCID: PMC5382463. 28381231 PMC5382463

[31] Park JH, Spiegelman DL, Gold DR, Burge HA, Milton DK. Predictors of airborne endotoxin in the home. Environ Health Perspect. 2001;109(8):859–64. doi: 10.1289/ehp.01109859 ; PubMed Central PMCID: PMC1240416. 11564624 PMC1240416

[32] Heinrich J, Gehring U, Douwes J, Koch A, Fahlbusch B, Bischof W, et al. Pets and vermin are associated with high endotoxin levels in house dust. Clin Exp Allergy. 2001;31(12):1839–45. doi: 10.1046/j.1365-2222.2001.01220.x . 11737034

[33] Sampson HA. Role of immediate food hypersensitivity in the pathogenesis of atopic dermatitis. J Allergy Clin Immunol. 1983;71(5):473–80. doi: 10.1016/0091-6749(83)90464-5 . 6841827

[34] Shoda T, Futamura M, Yang L, Yamamoto-Hanada K, Narita M, Saito H, et al. Timing of eczema onset and risk of food allergy at 3 years of age: A hospital-based prospective birth cohort study. J Dermatol Sci. 2016;84(2):144–8. Epub 20160802. doi: 10.1016/j.jdermsci.2016.08.003 . 27523805

[35] Thorsteinsdottir S, Thyssen JP, Stokholm J, Vissing NH, Waage J, Bisgaard H. Domestic dog exposure at birth reduces the incidence of atopic dermatitis. Allergy. 2016;71(12):1736–44. Epub 20160809. doi: 10.1111/all.12980 . 27385647

