# Supplementary material for: Associations between fetal or infancy pet exposure and food allergies: The Japan Environment and Children’s Study
Source: PLoS One. 2023 Mar 29;18(3):e0282725. doi: 10.1371/journal.pone.0282725 (PMC10057762; doi:10.1371/journal.pone.0282725)
Supplement: S1 Table — (DOCX) [file pone.0282725.s001.docx]

**Supplementary material**

**S1 Table.** Questionnaire Content

| **Questionnaire** | **Respondent** | **Response period** | **Question** | **Answer** |
| --- | --- | --- | --- | --- |
| M-T1 | Pregnant women | In the first trimester | Please check out who currently lives with you. | Child(ren) |
| M-T1 | Pregnant women | in the first trimester | Please check any of the diseases listed below that you have been diagnosed with by a physician from birth until today. | Bronchial asthma, allergic rhinitis, pollinosis, atopic dermatitis, allergic conjunctivitis, and food allergy |
| M-T2 | Pregnant women | In the second/third trimesters | Please check your smoking status. | "Never," "Previously did, but quit before recognizing current pregnancy", "Previously did, but quit after finding out current pregnancy," or "I still smoke." |
| M-T2 | Pregnant women | In the second/third trimesters | Please check the frequency of cleaning the floor of the living room with a vacuum cleaner (average throughout the year). | "Everyday," "A few times a week," "Once a week," "1-2 times a month", "A few times a year," or "Rarely or never" |
| M-T2 | Pregnant women | In the second/third trimesters | Do you currently have a pet in your home?  If so, what kind? | Yes or No.  Cat, Bird, Dog (kept inside of residence), Dog (kept outside of residence), Hamster, Tortoise, and Other pet |
| M-T2 | Pregnant women | In the second/third trimesters | Please indicate your highest level of education. (check only one) | "Junior high school," "High school," "Technical junior college," "Technical/vocational college," "Associate degree," "Bachelor’s degree," or "Graduate degree(Master’s/Doctor’s)" |
| M-T2 | Pregnant women | In the second/third trimesters | Please indicate your partner's highest level of education. (check only one) | "Junior high school," "High school," "Technical junior college," "Technical/vocational college," "Associate degree," "Bachelor’s degree," or "Graduate degree(Master’s/Doctor’s)" |
| M-T2 | Pregnant women | In the second/third trimesters | Please indicate your annual household income. (check only one) | "Less than 2 million yen", "2 million yen to less than 4 million yen", "4 million yen to less than 6 million yen", "6 million yen to less than 8 million yen", "8 million yen to less than 10 million yen", "10 million yen to less than 12 million yen", "12 million yen to less than 15 million yen", "15 million yen to less than 20 million yen", or "20 million yen and over" |
| C-6M | Caregivers | When the children were 6 months of age | Has your child begun attending a childcare facility (daycare center/nursery)? | Yes or No. |
| C-6M | Caregivers | When the children were 6 months of age | From the time your child started living at home (after birth discharge) to the present, have you kept animals (pets or domestic animals) in your home? If yes, please select all that you had (or have). | Yes or No.  Cat, Dog (kept inside of residence), Dog (kept outside of residence), cow, horse, pig, hen, and Other pet |
| C-1Y | Caregivers | When the children were 1 year of age | Has your child ever been diagnosed by a physician with any of the following diseases? If yes, please select all. | Atopic dermatitis and food allergy. |
| C-1hY | Caregivers | When the children were 1.5 years of age | Has your child ever been diagnosed by a physician with any of the following diseases since age 1 ((Including any ongoing hospital visits or treatment))? If yes, please select all | atopic dermatitis and food allergy. |
| C-1hY | Caregivers | When the children were 1.5 years of age | Please answer the consumption status of specific foods (egg, milk, wheat, soybean, fish, rice, fruit, crustacean, soba, sesame, and nut). | "no avoidance (eating normally)", "has never eaten", "partial avoidance", "has eaten before, however, now complete avoidance." |
| C-1hY | Caregivers | When the children were 1.5 years of age | Did your child have abnormal blood or skin test results with a specific food (egg, milk, wheat, soybean, fish, rice, fruit, crustacean, soba, sesame, and nut).? | Yes or No (egg, milk, wheat, soybean, fish, rice, fruit, crustacean, soba, sesame, and nut, respectively). |
| C-1hY | Caregivers | When the children were 1.5 years of age | Did your child have allergic symptoms after eating specific food (egg, milk, wheat, soybean, fish, rice, fruit, crustacean, soba, sesame, and nut).? | Yes or No (egg, milk, wheat, soybean, fish, rice, fruit, crustacean, soba, sesame, and nut, respectively). |
| C-2Y | Caregivers | When the children were 2 years of age | Has your child ever been diagnosed by a physician with any of the following diseases since the age of 1.5 ((Including any ongoing hospital visits or treatment))? If yes, please select all | atopic dermatitis and food allergy. |
| C-2Y | Caregivers | When the children were 2 years of age | Please answer the consumption status of specigic foods (egg, milk, wheat, soybean, fish, rice, fruit, crustacean, soba, sesame, and nut). | "no avoidance (eating normally)," "has never eaten," "partial avoidance," "has eaten before, however, now complete avoidance." |
| C-2Y | Caregivers | When the children were 2 years of age | Did your child have abnormal blood or skin test results with a specific food (egg, milk, wheat, soybean, fish, rice, fruit, crustacean, soba, sesame, and nut).? | Yes or No (egg, milk, wheat, soybean, fish, rice, fruit, crustacean, soba, sesame, and nut, respectively). |
| C-2Y | Caregivers | When the children were 2 years of age | Did your child have allergic symptoms after eating specific food (egg, milk, wheat, soybean, fish, rice, fruit, crustacean, soba, sesame, and nut).? | Yes or No (egg, milk, wheat, soybean, fish, rice, fruit, crustacean, soba, sesame, and nut, respectively). |
| C-3Y | Caregivers | When the children were 3 years of age | Has your child ever been diagnosed by a physician with any of the following diseases since the age of 2 ((Including any ongoing hospital visits or treatment))? If yes, please select all | atopic dermatitis and food allergy. |
| C-3Y | Caregivers | When the children were 3 years of age | Please answer the consumption status of specific foods (egg, milk, wheat, soybean, fish, rice, fruit, crustacean, soba, sesame, and nut). | "no avoidance (eating normally)," "has never eaten," "partial avoidance," "has eaten before, however, now complete avoidance." |
| C-3Y | Caregivers | When the children were 3 years of age | Did your child have abnormal blood or skin test results with a specific food (egg, milk, wheat, soybean, fish, rice, fruit, crustacean, soba, sesame, and nut).? | Yes or No (egg, milk, wheat, soybean, fish, rice, fruit, crustacean, soba, sesame, and nut, respectively). |
| C-3Y | Caregivers | When the children were 3 years of age | Did your child have allergic symptoms after eating specific food (egg, milk, wheat, soybean, fish, rice, fruit, crustacean, soba, sesame, and nut).? | Yes or No (egg, milk, wheat, soybean, fish, rice, fruit, crustacean, soba, sesame, and nut, respectively). |
