# Supplementary material for: Associations between fetal or infancy pet exposure and food allergies: The Japan Environment and Children’s Study
Source: PLoS One. 2023 Mar 29;18(3):e0282725. doi: 10.1371/journal.pone.0282725 (PMC10057762; doi:10.1371/journal.pone.0282725)
Supplement: S2 Table — (DOCX) [file pone.0282725.s002.docx]

**Supplementary material**

**S2 Table.** Collinearity of the selected covariates

| Cramér's V | | | |  |
| --- | --- | --- | --- | --- |
|  | Unit region | Older siblings | Maternal history of allergic disease |  |
| Older siblings | 0.043 |  |  |  |
| Maternal history of allergic disease | 0.045 | 0.007 |  |  |
| Maternal smoking | 0.048 | 0.022 | −0.013 |  |
|  |  |  |  |  |
| Quartile-point correlation coefficient (φ coefficient) | | |  |  |
|  | Older siblings | Maternal history of allergic disease |  |  |
| Older siblings |  |  |  |  |
| Maternal history of allergic disease | 0.011 |  |  |  |
| Maternal smoking | 0.067 | −0.039 |  |  |
|  |  |  |  |  |
| Correlation ratio (η^2) | | | | |
|  | Unit region | Older siblings | Maternal history of allergic disease | Maternal smoking |
| Highest level of maternal education | 0.002 | 0.003 | 0.003 | 0.038 |
| Highest level of paternal education | 0.001 | 0.003 | 0.002 | 0.026 |
| Annual household income | 0.001 | 0 | 0.001 | 0.007 |
| Maternal age | 0.001 | 0.048 | 0.002 | 0.003 |
|  |  |  |  |  |
| Spearman's correlation coefficient | | | |  |
|  | Highest level of maternal education | Highest level of paternal education | Annual household income |  |
| Highest level of maternal education | 0.417 |  |  |  |
| Annual household income | 0.286 | 0.265 |  |  |
| Maternal age | 0.164 | 0.149 | 0.234 |  |
