# Supplementary material for: Associations between fetal or infancy pet exposure and food allergies: The Japan Environment and Children’s Study
Source: PLoS One. 2023 Mar 29;18(3):e0282725. doi: 10.1371/journal.pone.0282725 (PMC10057762; doi:10.1371/journal.pone.0282725)
Supplement: S3 Table — (DOCX) [file pone.0282725.s003.docx]

**Supplementary material**

**S3 Table.** Association between pet exposure and incidence risk of specific food allergy until age 3 years

|  |  | Egg allergy | | | | | |
| --- | --- | --- | --- | --- | --- | --- | --- |
|  |  | IP (C) | IP (E) | cOR | (95% CI) | aOR | (95% CI) |
| Any pet | F | 8.43% | 7.24% | 0.85 | (0.79–0.90) | 0.90 | (0.83–0.96) |
| Dog kept indoor | F | 8.29% | 6.94% | 0.82 | (0.74–0.91) | 0.84 | (0.75–0.93) |
| Dog kept indoor | I | 8.30% | 7.13% | 0.85 | (0.77–0.93) | 0.84 | (0.76–0.92) |
| Dog kept outdoor | F | 8.21% | 7.19% | 0.87 | (0.74–1.00) | 0.97 | (0.83–1.13) |
| Dog kept outdoor | I | 8.22% | 7.05% | 0.85 | (0.73–0.97) | 0.92 | (0.79–1.06) |
| Cat | F | 8.29% | 6.46% | 0.76 | (0.67–0.86) | 0.83 | (0.72–0.94) |
| Cat | I | 8.31% | 6.52% | 0.77 | (0.68–0.86) | 0.82 | (0.72–0.91) |
| Turtle | F | 8.19% | 6.74% | 0.81 | (0.60–1.08) | 0.90 | (0.67–1.20) |
| Hamster | F | 8.18% | 7.28% | 0.88 | (0.64–1.19) | 0.93 | (0.68–1.26) |
| Bird | F | 8.17% | 9.24% | 1.15 | (0.83–1.56) | 1.17 | (0.85–1.60) |
|  |  |  |  |  |  |  |  |
|  |  | Milk allergy | | | | | |
|  |  | IP (C) | IP (E) | cOR | (95% CI) | aOR | (95% CI) |
| Any pet | F | 2.95% | 2.40% | 0.81 | (0.72–0.91) | 0.90 | (0.79–1.01) |
| Dog kept indoor | F | 2.89% | 2.18% | 0.75 | (0.62–0.90) | 0.82 | (0.68–0.98) |
| Dog kept indoor | I | 2.90% | 2.28% | 0.78 | (0.66–0.92) | 0.84 | (0.70–0.98) |
| Dog kept outdoor | F | 2.86% | 2.13% | 0.74 | (0.56–0.97) | 0.82 | (0.62–1.08) |
| Dog kept outdoor | I | 2.88% | 1.82% | 0.63 | (0.47–0.82) | 0.69 | (0.52–0.90) |
| Cat | F | 2.88% | 2.16% | 0.75 | (0.60–0.92) | 0.88 | (0.70–1.08) |
| Cat | I | 2.88% | 2.22% | 0.76 | (0.63–0.92) | 0.89 | (0.73–1.07) |
| Turtle | F | 2.83% | 2.96% | 1.05 | (0.68–1.60) | 1.16 | (0.75–1.78) |
| Hamster | F | 2.83% | 3.15% | 1.12 | (0.70–1.76) | 1.23 | (0.77–1.94) |
| Bird | F | 2.84% | 2.31% | 0.81 | (0.44–1.47) | 0.89 | (0.48–1.63) |
|  |  |  |  |  |  |  |  |
|  |  | Wheat allergy | | | | | |
|  |  | IP (C) | IP (E) | cOR | (95% CI) | aOR | (95% CI) |
| Any pet | F | 0.95% | 0.73% | 0.76 | (0.61–0.94) | 0.82 | (0.66–1.01) |
| Dog kept indoor | F | 0.93% | 0.65% | 0.70 | (0.49–0.97) | 0.73 | (0.52–1.01) |
| Dog kept indoor | I | 0.92% | 0.76% | 0.83 | (0.62–1.10) | 0.86 | (0.64–1.13) |
| Dog kept outdoor | F | 0.91% | 0.71% | 0.78 | (0.48–1.24) | 0.88 | (0.54–1.40) |
| Dog kept outdoor | I | 0.92% | 0.62% | 0.67 | (0.42–1.07) | 0.75 | (0.46–1.19) |
| Cat | F | 0.93% | 0.45% | 0.48 | (0.30–0.76) | 0.54 | (0.34–0.85) |
| Cat | I | 0.93% | 0.53% | 0.57 | (0.38–0.83) | 0.63 | (0.42–0.92) |
| Turtle | F | 0.90% | 1.21% | 1.35 | (0.69–2.62) | 1.50 | (0.77–2.91) |
| Hamster | F | 0.90% | 0.99% | 1.10 | (0.49–2.47) | 1.19 | (0.52–2.67) |
| Bird | F | 0.90% | 0.84% | 0.93 | (0.34–2.49) | 0.99 | (0.36–2.64) |
|  |  |  |  |  |  |  |  |
|  |  | Soybean allergy | | | | | |
|  |  | IP (C) | IP (E) | cOR | (95% CI) | aOR | (95% CI) |
| Any pet | F | 0.42% | 0.35% | 0.82 | (0.60–1.12) | 0.92 | (0.67–1.25) |
| Dog kept indoor | F | 0.42% | 0.30% | 0.71 | (0.43–1.15) | 0.77 | (0.47–1.26) |
| Dog kept indoor | I | 0.42% | 0.33% | 0.80 | (0.51–1.22) | 0.85 | (0.55–1.30) |
| Dog kept outdoor | F | 0.42% | 0.20% | 0.47 | (0.19–1.14) | 0.55 | (0.22–1.34) |
| Dog kept outdoor | I | 0.42% | 0.24% | 0.58 | (0.27–1.22) | 0.66 | (0.31–1.41) |
| Cat | F | 0.42% | 0.24% | 0.57 | (0.30–1.06) | 0.67 | (0.35–1.26) |
| Cat | I | 0.43% | 0.16% | 0.37 | (0.18–0.73) | 0.43 | (0.21–0.86) |
| Turtle | F | 0.40% | 0.67% | 1.67 | (0.68–4.05) | 1.85 | (0.76–4.51) |
| Hamster | F | 0.41% | 0.33% | 0.81 | (0.20–3.26) | 0.91 | (0.22–3.66) |
| Bird | F | 0.41% | 0% |  |  |  |  |
|  |  |  |  |  |  |  |  |
|  |  | Fish allergy | | | | | |
|  |  | IP (C) | IP (E) | cOR | (95% CI) | aOR | (95% CI) |
| Any pet | F | 0.47% | 0.41% | 0.86 | (0.64–1.14) | 0.91 | (0.67–1.21) |
| Dog kept indoor | F | 0.47% | 0.30% | 0.63 | (0.38–1.02) | 0.69 | (0.41–1.12) |
| Dog kept indoor | I | 0.48% | 0.29% | 0.61 | (0.38–0.95) | 0.65 | (0.40–1.02) |
| Dog kept outdoor | F | 0.45% | 0.59% | 1.32 | (0.78–2.21) | 1.33 | (0.78–2.25) |
| Dog kept outdoor | I | 0.45% | 0.55% | 1.22 | (0.73–2.02) | 1.21 | (0.72–2.01) |
| Cat | F | 0.46% | 0.33% | 0.72 | (0.41–1.22) | 0.74 | (0.43–1.27) |
| Cat | I | 0.46% | 0.35% | 0.76 | (0.47–1.22) | 0.78 | (0.48–1.26) |
| Turtle | F | 0.46% | 0.54% | 1.19 | (0.44–3.18) | 1.26 | (0.46–3.40) |
| Hamster | F | 0.46% | 0.33% | 0.72 | (0.17–2.91) | 0.78 | (0.19–3.13) |
| Bird | F | 0.46% | 0.42% | 0.92 | (0.22–3.70) | 0.97 | (0.23–3.90) |
|  |  |  |  |  |  |  |  |
|  |  | Rice allergy | | | | | |
|  |  | IP (C) | IP (E) | cOR | (95% CI) | aOR | (95% CI) |
| Any pet | F | 0.04% | 0.02% | 0.57 | (0.16–1.93) | 0.72 | (0.20–2.44) |
| Dog kept indoor | F | 0.03% | 0.03% | 1.06 | (0.24–4.52) | 1.30 | (0.30–5.64) |
| Dog kept indoor | I | 0.03% | 0.03% | 0.86 | (0.19–3.65) | 1.03 | (0.23–4.45) |
| Dog kept outdoor | F | 0.03% | 0.04% | 1.20 | (0.16–8.91) | 1.76 | (0.23–13.34) |
| Dog kept outdoor | I | 0.03% | 0.07% | 2.18 | (0.50–9.32) | 3.15 | (0.71–13.8) |
| Cat | F | 0.04% | 0% |  |  |  |  |
| Cat | I | 0.04% | 0% |  |  |  |  |
| Turtle | F | 0.03% | 0% |  |  |  |  |
| Hamster | F | 0.03% | 0% |  |  |  |  |
| Bird | F | 0.03% | 0% |  |  |  |  |
|  |  |  |  |  |  |  |  |
|  |  | Fruit allergy | | | | | |
|  |  | IP (C) | IP (E) | cOR | (95% CI) | aOR | (95% CI) |
| Any pet | F | 0.53% | 0.42% | 0.80 | (0.60–1.05) | 0.89 | (0.67–1.18) |
| Dog kept indoor | F | 0.51% | 0.40% | 0.78 | (0.51–1.19) | 0.88 | (0.57–1.35) |
| Dog kept indoor | I | 0.50% | 0.50% | 1.00 | (0.70–1.42) | 1.12 | (0.78–1.59) |
| Dog kept outdoor | F | 0.50% | 0.47% | 0.94 | (0.52–1.67) | 1.08 | (0.60–1.93) |
| Dog kept outdoor | I | 0.51% | 0.34% | 0.67 | (0.35–1.26) | 0.77 | (0.40–1.44) |
| Cat | F | 0.51% | 0.33% | 0.65 | (0.37–1.10) | 0.75 | (0.43–1.27) |
| Cat | I | 0.52% | 0.33% | 0.64 | (0.39–1.05) | 0.74 | (0.45–1.21) |
| Turtle | F | 0.51% | 0.27% | 0.53 | (0.13–2.13) | 0.61 | (0.15–2.45) |
| Hamster | F | 0.51% | 0.17% | 0.33 | (0.04–2.32) | 0.38 | (0.05–2.74) |
| Bird | F | 0.50% | 0.63% | 1.26 | (0.40–3.93) | 1.38 | (0.43–4.31) |
|  |  |  |  |  |  |  |  |
|  |  | Crustacean allergy | | | | | |
|  |  | IP (C) | IP (E) | cOR | (95% CI) | aOR | (95% CI) |
| Any pet | F | 0.60% | 0.55% | 0.92 | (0.71–1.17) | 0.97 | (0.75–1.24) |
| Dog kept indoor | F | 0.59% | 0.49% | 0.82 | (0.55–1.21) | 0.85 | (0.57–1.25) |
| Dog kept indoor | I | 0.60% | 0.46% | 0.77 | (0.53–1.10) | 0.78 | (0.54–1.13) |
| Dog kept outdoor | F | 0.58% | 0.59% | 1.01 | (0.60–1.70) | 1.08 | (0.64–1.82) |
| Dog kept outdoor | I | 0.59% | 0.38% | 0.64 | (0.34–1.15) | 0.67 | (0.36–1.22) |
| Cat | F | 0.59% | 0.45% | 0.76 | (0.47–1.20) | 0.84 | (0.52–1.33) |
| Cat | I | 0.60% | 0.41% | 0.69 | (0.44–1.06) | 0.75 | (0.48–1.17) |
| Turtle | F | 0.59% | 0.27% | 0.46 | (0.11–1.83) | 0.49 | (0.12–1.96) |
| Hamster | F | 0.58% | 0.66% | 1.14 | (0.42–3.05) | 1.18 | (0.43–3.16) |
| Bird | F | 0.58% | 0.63% | 1.08 | (0.34–3.37) | 1.15 | (0.36–3.59) |
|  |  |  |  |  |  |  |  |
|  |  | Soba allergy | | | | | |
|  |  | IP (C) | IP (E) | cOR | (95% CI) | aOR | (95% CI) |
| Any pet | F | 0.46% | 0.40% | 0.86 | (0.64–1.14) | 0.93 | (0.69–1.24) |
| Dog kept indoor | F | 0.46% | 0.33% | 0.72 | (0.45–1.14) | 0.79 | (0.49–1.26) |
| Dog kept indoor | I | 0.46% | 0.36% | 0.78 | (0.51–1.17) | 0.85 | (0.56–1.28) |
| Dog kept outdoor | F | 0.46% | 0.28% | 0.60 | (0.28–1.27) | 0.64 | (0.30–1.36) |
| Dog kept outdoor | I | 0.46% | 0.28% | 0.60 | (0.29–1.21) | 0.63 | (0.31–1.28) |
| Cat | F | 0.45% | 0.43% | 0.95 | (0.58–1.52) | 1.04 | (0.64–1.67) |
| Cat | I | 0.45% | 0.41% | 0.91 | (0.58–1.41) | 0.99 | (0.63–1.55) |
| Turtle | F | 0.45% | 0.67% | 1.51 | (0.62–3.66) | 1.66 | (0.68–4.03) |
| Hamster | F | 0.45% | 0.17% | 0.36 | (0.05–2.60) | 0.41 | (0.05–2.95) |
| Bird | F | 0.45% | 0.84% | 1.89 | (0.70–5.08) | 1.99 | (0.73–5.37) |
|  |  |  |  |  |  |  |  |
|  |  | Sesame allergy | | | | | |
|  |  | IP (C) | IP (E) | cOR | (95% CI) | aOR | (95% CI) |
| Any pet | F | 0.30% | 0.23% | 0.76 | (0.52–1.10) | 0.88 | (0.60–1.28) |
| Dog kept indoor | F | 0.30% | 0.16% | 0.52 | (0.26–1.02) | 0.60 | (0.30–1.18) |
| Dog kept indoor | I | 0.30% | 0.20% | 0.68 | (0.39–1.17) | 0.76 | (0.43–1.31) |
| Dog kept outdoor | F | 0.29% | 0.12% | 0.40 | (0.12–1.26) | 0.47 | (0.14–1.47) |
| Dog kept outdoor | I | 0.30% | 0.10% | 0.35 | (0.11–1.09) | 0.39 | (0.12–1.23) |
| Cat | F | 0.28% | 0.33% | 1.17 | (0.67–2.02) | 1.42 | (0.81–2.45) |
| Cat | I | 0.29% | 0.29% | 1.03 | (0.60–1.74) | 1.22 | (0.71–2.08) |
| Turtle | F | 0.29% | 0.40% | 1.42 | (0.45–4.44) | 1.66 | (0.52–5.22) |
| Hamster | F | 0.29% | 0% |  |  |  |  |
| Bird | F | 0.29% | 0.21% | 0.73 | (0.10–5.22) | 0.80 | (0.11–5.76) |
|  |  |  |  |  |  |  |  |
|  |  | Nut allergy | | | | | |
|  |  | IP (C) | IP (E) | cOR | (95% CI) | aOR | (95% CI) |
| Any pet | F | 0.98% | 0.95% | 0.97 | (0.80–1.17) | 1.04 | (0.85–1.25) |
| Dog kept indoor | F | 1.00% | 0.70% | 0.70 | (0.50–0.96) | 0.75 | (0.53–1.02) |
| Dog kept indoor | I | 1.00% | 0.69% | 0.69 | (0.51–0.92) | 0.72 | (0.53–0.97) |
| Dog kept outdoor | F | 0.97% | 1.11% | 1.15 | (0.78–1.67) | 1.20 | (0.81–1.75) |
| Dog kept outdoor | I | 0.96% | 1.14% | 1.18 | (0.83–1.67) | 1.22 | (0.85–1.74) |
| Cat | F | 0.98% | 0.88% | 0.90 | (0.64–1.25) | 0.97 | (0.69–1.35) |
| Cat | I | 0.98% | 0.90% | 0.92 | (0.68–1.24) | 0.99 | (0.73–1.34) |
| Turtle | F | 0.97% | 1.35% | 1.40 | (0.74–2.62) | 1.50 | (0.79–2.81) |
| Hamster | F | 0.96% | 1.66% | 1.73 | (0.92–3.24) | 1.93 | (1.02–3.62) |
| Bird | F | 0.97% | 1.47% | 1.53 | (0.72–3.23) | 1.59 | (0.74–3.36) |
